# Roles of Oxygen and Hydrogen in Crystal Orientation Transition of Copper Foils for High-Quality Graphene Growth

**DOI:** 10.1038/srep45358

**Published:** 2017-04-03

**Authors:** Junxiong Hu, Jianbao Xu, Yanfei Zhao, Lin Shi, Qi Li, Fengkui Liu, Zaka Ullah, Weiwei Li, Yufen Guo, Liwei Liu

**Affiliations:** 1 Key Lab of Nanodevices and Applications, Suzhou Institute of Nano-Tech and Nano-Bionics, Chinese Academy of Sciences (CAS), Suzhou, 215123, P. R. China; 2 Department of Physics, Institute of Low-dimensional Carbons and Device Physics, Shanghai University, Shanghai, 200444, P. R. China; 3 College of Materials Sciences and Opto-Electronic Technology, University of Chinese Academy of Sciences, Beijing, 100049, P. R. China; 4 Nano-X, Suzhou Institute of Nano-Tech and Nano-Bionics, Chinese Academy of Sciences (CAS), Suzhou, 215123, P. R. China; 5 Platform for Characterization &Test, Suzhou Institute of Nano-Tech and Nano-Bionics, Chinese Academy of Sciences (CAS), Suzhou, 215123, P. R. China; 6 Suzhou Graphene Nanotechnology Co., Ltd., Suzhou, 215123, P. R. China

## Abstract

The high-quality graphene film can be grown on single-crystal Cu substrate by seamlessly stitching the aligned graphene domains. The roles of O_2_ and H_2_ have been intensively studied in the graphene growth kinetics, including lowering the nucleation sites and tailoring the domain structures. However, how the O_2_ and H_2_ influence Cu orientations during recrystallization prior to growing graphene, still remains unclear. Here we report that the oxidation of Cu surface tends to stabilize the Cu(001) orientation while impedes the evolution of Cu(111) single domain during annealing process. The crystal orientation-controlled synthesis of aligned graphene seeds is further realized on the long-range ordered Cu(111) substrate. With decreasing the thickness of oxide layer on Cu surface by introducing H_2_, the Cu(001) orientation changes into Cu(111) orientation. Meanwhile, the average domain size of Cu foils is increased from 50 μm to larger than 1000 μm. The density functional theory calculations reveal that the oxygen increases the energy barrier for Cu(111) surface and makes O/Cu(001) more stable than O/Cu(111) structure. Our work can be helpful for revealing the roles of O_2_ and H_2_ in controlling the formation of Cu single-crystal substrate as well as in growing high-quality graphene films.

Chemical vapor deposition (CVD) method on Cu substrates has emerged as a promising technique for realizing high-quality^1^ and large-scale^2^ graphene films, which can be scaled up to meter in size by employing roll-to-roll (R2R) method^3^. However, CVD-grown graphene films are usually polycrystalline and consist of numerous grains separated by grain boundaries^4^,^5^, which remarkably degrade their electrical and mechanical properties^6^,^7^. In order to suppress the grain boundaries, an efficient approach is found to align the graphene domain orientation on a single-crystalline catalyst surface, and then seamlessly coalesce adjacent aligned islands to form uniform monocrystalline graphene. Various efforts have been made to achieve the well-ordered substrates, including the use of expensive single-crystal Ge(110)^8^, Ni(111)^9^ and Cu(111)^10^. On the contrary, thermal recrystallization in commercial polycrystalline Cu is a versatile way to produce long-range ordered substrate. For instance, the aligned graphene can be grown on the large Cu(111) grain annealed by several hours^11^. By further polishing the surface, the seamless stitching was realized by merging the neighbor graphene domains^12^. Recently, the aligned square domains were also grown on single-crystal Cu(001) surface^13^. In these studies, a precise control of long-range crystalline in Cu substrates is critical for obtaining the well-aligned graphene domains.

For the roles of O_2_ and H_2_ in the graphene growth kinetics, previous study reported that the O_2_ can lower nucleation density^14^,^15^,^16^ and reduce the energy barrier for carbon precursor attachment to the edges of graphene^14^,^17^, thus the growth rate of single-crystal graphene can be increased^18^. Besides, the role of H_2_ has been reported to act as an etching reagent that tailors the domain structure^19^,^20^ and terminates the graphene edges to form few layers^21^,^22^. Therefore, the study of the roles of O_2_ and H_2_ in the crystal orientation transition of underlying substrate is a prerequisite for achieving precise control of substrate for single-crystalline graphene growth^23^,^24^.

In this work, we designed 3 schemes to control the degree of oxidation by introducing H_2_ at different heat treatment stages, obtaining 3 different thicknesses of oxidation layers measured by X-ray photoelectron spectroscopy (XPS), to study how the Cu oxides affects the substrate orientation as well as grain size. In order to reveal the role of O_2_ in crystal orientation transition of Cu foils, the density functional theory (DFT) calculations were conducted to study the effect of O_2_ on the stabilities of Cu(001) and Cu(111). After obtaining the Cu(111) single domain, the well-aligned hexagonal graphene domains showing similar orientations were grown by ambient pressure CVD(APCVD). Understanding the roles of O_2_ and H_2_ in the crystal orientation transition of Cu foil is beneficial for the growth of well-aligned graphene arrays on single domain Cu foils. This work using the roll to roll or batch to batch process can deliver scalable production of high-quality graphene films.

## Results

### Characterizations of the evolution of single domain Cu(111)

**Figure 1:**
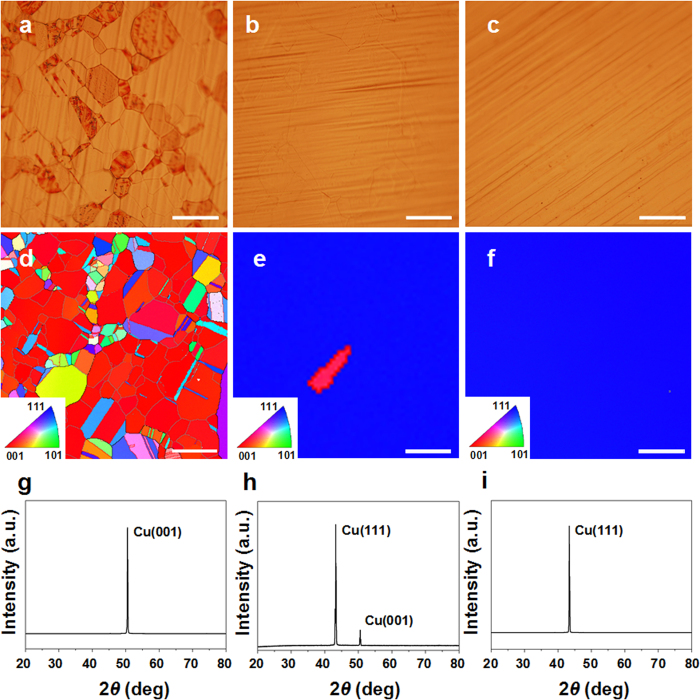
Surface morphology and structure characterizations of Cu foils annealed under 3 schemes. Scheme 1: heating and annealing under Ar flow. Scheme 2: heating and annealing under H_2_ and Ar flows, respectively. Scheme 3: heating and annealing under H_2_ flow. All the samples were heated up to 1040 °C under ambient pressure within 40 min, then annealed at 1040 °C for 30 min. (**a**–**c**) Optical images of Cu foils annealed under scheme 1–3. Scale bars, 50 μm. (**d**–**f**) EBSD orientation maps of Cu foils annealed under scheme 1–3. The left inset in (**d**–**f**) shows the corresponding inverse pole figure for each map displayed. Grains marked blue are oriented along plane Cu(111) while grains in red are along Cu(001). Scale bars, 200 μm (**d**,**e**); 500 μm (**f**). (**g**–**i**) XRD profiles of Cu foils annealed under scheme 1–3. The measurements of EBSD and XRD were made from the same area of Cu foils.

During thermal treatments of Cu foils in the tube furnace, the main source of oxygen mostly can be divided into three parts: (a) the trapped air in quartz tube; (b) the native oxide layer on Cu surface; (c) the impure gas source and tiny leakages from environment. According to the sources of oxygen, we designed 3 schemes to control the degree of oxidation through introducing H_2_ at different heat treatment stages. The corresponding orientations and grain sizes of Cu foils annealed under 3 schemes are shown in Fig. 1. Before heating, the purging process was conducted to minimize the trapped air in CVD chamber (see method). In scheme 1, in order to introduce relatively high level of oxygen, only the Ar gas without H_2_ was introduced during heating and annealing, and in this scheme the Cu foil was subjected to the above mentioned three sources of oxygen. Figure 1a shows a layer of mosaic patterns formed on Cu surface. Electron backscatter diffraction (EBSD) map shows that the Cu surface owns many randomly oriented grains with size 50–100 μm (Fig. 1d), while the X-ray diffraction (XRD) reveals that the Cu has only Cu(001) orientation (Fig. 1g). The difference between EBSD and XRD results can be attributed to the different penetration depths. XRD can probe the depth of micrometers while EBSD just reveals the information from tens of nanometers thickness of Cu surface^25^. When the quartz-tube was not purged, the grain size of Cu foil was further reduced to 5–10 μm (Supplementary Fig. S1). In scheme 2, in order to introduce the medium level of oxygen, we introduced H_2_ during heating. However, annealing was still performed under Ar flow. In this scheme, the trapped air and native oxide layer on Cu surface can be removed and the oxygen sources only came from impure gas and tiny leakages. Figure 1b shows that the mosaic patterns on Cu surface start to disappear. EBSD map (Fig. 1e) shows that most of the orientations in Fig. 1d have been transformed to Cu(111) plane. The Cu(111) orientation is further confirmed by XRD profile (Fig. 1h), as a main peak at ~42° is assigned to Cu(111) diffraction. Meanwhile, the grain size increases from 50 μm (scheme 1) to 1000 μm (scheme 2).

In scheme 3, in order to completely suppress the oxidation, the H_2_ was introduced in the processes of both heating and annealing. EBSD image shows that the Cu surface has become single domain Cu(111) and been free from grain boundaries, in the meantime, grain size increases to larger than 1000 μm (Fig. 1f) (Supplementary Fig. S2). The XRD analysis also reveals the single Cu(111) oriented grain (Fig. 1i). Furthermore, the Cu(111) orientation can be obtained in 1 min (Supplementary Fig. S3e,f). However, without introduction of H_2_ flow (under Ar atmosphere), even though the annealing time was extended to 180 min, the Cu foil still remained in Cu(001) orientation. Nevertheless, followed by introducing H_2_ for 1 min, surprisingly, the Cu(111) orientation can be obtained again (Supplementary Fig. S3a–d). The combined analysis of EBSD and XRD clearly proves that the introduction of H_2_ tends to assist the evolution of Cu(111) orientation.

In order to reliably achieve single domain Cu(111), other thermodynamics parameters such as temperature, pressure and cooling rate were also investigated (Supplementary Fig. S4). The high temperature up to 1040 °C, near to melting point of Cu foil (1088 °C), was necessary to obtain Cu(111) orientation^11^,^23^,^24^. However, when the quartz-tube pressure was decreased to 150 Pa, the Cu(111) orientation can be achieved at 1010 °C, indicating that the decreasing pressure can reduce the orientation-transition temperature for Cu(111)^26^,^27^. In addition, when the cooling rate was maintained at 0.3 °C/s, the XRD profiles show that apart from Cu(111), other phases such as Cu(001) and Cu(220) will also precipitate upon cooling. Once the cooling rate exceeds 0.9 °C/s, the impure phases can be suppressed efficiently, indicating that fast cooling was in favor of Cu(111) evolution^28^.

### Characterizations of the aligned graphene domains and continuous film grown on single domain Cu(111)

**Figure 2:**
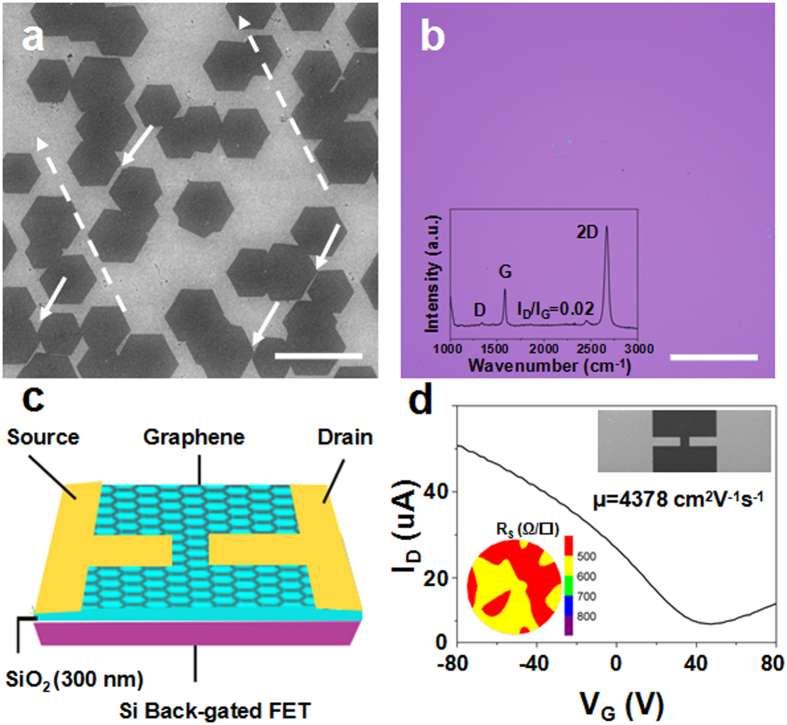
Optical and electrical characterizations of graphene grown on singe-domain Cu(111) substrate. (**a**) Scanning electron microscope (SEM) image of a partial-coverage aligned graphene seeds on SiO_2_/Si, where the white dashed lines indicate the orientations of graphene and the arrows point out the coalescence of adjacent islands. Scale bar, 20 μm. (**b**) Optical image of continuous graphene. The inset shows a representative single-point Raman spectrum. The negligible D band indicates the growth of high-quality graphene film. Scale bar, 20 μm. (**c**) Schematic of two-point back-gated field-effect-transistor (FET) device. (**d**) Current − voltage (*I*_*D*_ − *V*_*G*_) curve for the continuous graphene grown on Cu(111)-FET measured at room temperature. The right inset shows the SEM image of the device and the left inset shows a representative sheet resistance distribution of graphene film transferred onto SiO_2_/Si (1.5 mm in diameter).

Based on the obtained single domain Cu(111) substrates, the graphene was further grown on the ordered Cu(111) substrate by ambient pressure CVD (APCVD)^29^,^30^, as shown in Fig. 2. In order to investigate the graphene growth at early stage, we intentionally limited the growth time to produce the individual grains (Fig. 2a). In this partial coverage graphene, the well-aligned hexagonal graphene arrays display the similar orientations, which are indicated by the white dashed arrows. The whole sample exhibits the same feature (Supplementary Fig. S5). When the two identical orientations of islands are merged together, which are indicated by the white arrows, the grain boundaries between adjacent islands may be completely suppressed by the commensurate stitching^12^, providing a reliable and efficient strategy for the synthesis of wafer-scale single-crystal graphene.

The uniformity of continuous graphene film was further evaluated by optical microscopy. Figure 2b shows an optical micrograph of full coverage of graphene film. The uniform color contrast of the optical image indicates that the graphene film has an excellent thickness uniformity^31^. The inset in Fig. 2b shows a typical Raman spectrum with peaks for monolayer graphene, including a 2D-band with a full width at half-maximum (FWHM) of ~36 cm^−1^ located at ~2680 cm^−1^. In addition, the *I*_*2D*_/*I*_*G*_ ratio is ~2.7. Both FWHM and *I*_*2D*_/*I*_*G*_ ratio are on the merits for monolayer graphene^32^. Moreover, the defect density *n*_*do*_ can be calculated from the empirical formula^33^:


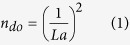



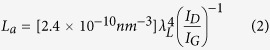


where *L*_α_ is the grain size and *λ*_*L*_ is the excitation laser wavelength (532.15 nm). According to the *I*_D_/*I*_G_ ratio (0.02), the grain size *L*_α_ is calculated to be 963 nm while the defect density *n*_*do*_ is calculated as 1.08 × 10^8^ cm^−2^, which shows a high-quality graphene^34^ (Supplementary Fig. S6). To further evaluate the electrical quality of graphene films grown on Cu(111), we fabricated back-gated field-effect transistors (FETs) on a highly doped p-type silicon substrate with 300 nm oxide. The schematic of two-probe FET with length and width channels each of 50 μm is shown in Fig. 2c. Figure 2d shows the transfer characteristic of source-drain current (*I*_*ds*_) versus gate voltage (*V*_*bg*_) at room temperature and the right inset gives the SEM image of fabricated FET device. The carrier mobility of graphene grown on Cu(111) obtained from the Drude formula (see method) is 4378 cm^2^ V^−1^ s^−1^, comparable with the mobility of single-crystal graphene^35^. The corresponding Dirac point is 42 V, which indicates a p-type behavior of graphene film^36^. In order to evaluate the electrical property of graphene in a wide area inspection, the sheet resistance map (50 points in an area of 15 × 15 mm^2^) of graphene film was measured by four-point probe measurement system (see method), as shown in the left inset of Fig. 2d. The sheet resistance of graphene grown on Cu(111) is around 500 Ω/□, showing an excellent electrical property^37^,^38^.

For comparative study, we also investigated graphene growth on polycrystalline Cu(001) surface (Supplementary Fig. S7). The monolayer graphene was also produced on Cu(001). However, the seed shapes are randomly formed, without the domain orientation preference^8^ (Fig. S7a). Even though there is no obvious contrast between the optical images of graphene grown on Cu(001) (Fig. S7b) and graphene grown on Cu(111) (Fig. 2b), the D-band of Raman spectra and the corresponding electrical properties show considerable difference. The *I*_*D*_/*I*_*G*_ ratio of graphene on Cu(001) is as large as 0.38 (Fig. S7b), with the defect density *n*_*do*_ as high as 8.53 × 10^10^ cm^−2^. Moreover, the carrier mobility is 870 cm^2 ^V^−1^ s^−1^ and the sheet resistance is 1000 Ω/□ (Fig. S7d). Therefore, the graphene electrical property grown on single domain Cu(111) is obviously superior to that grown on polycrystalline Cu(001)(Fig. S8). The electrical difference of the graphene films grown on Cu(111) and Cu(001) substrates would show different potential applications in electronic devices. For the graphene film grown on Cu(111), the excellent electrical quality of graphene film can be used for flexible transparent electrodes^2^,^3^. While the graphene film on Cu(001) has larger sheet resistance, such graphene can be used to fabricate strain sensors, as graphene with low conductivity delivers higher sensing performance^39^,^40^.

## Discussion

**Figure 3:**
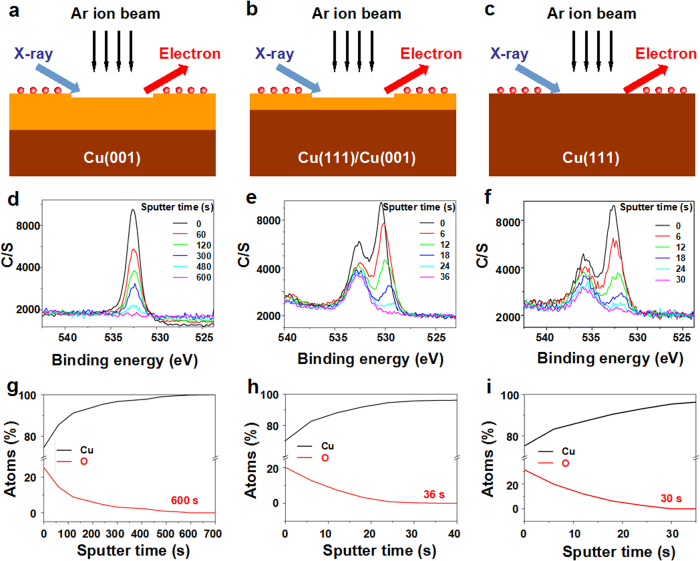
XPS analysis of the Cu foils annealed under 3 schemes. (**a**–**c**) Schematic structures for the XPS depth-profiling of three different thicknesses of copper oxides on corresponding substrate orientations. The orange layer refers to the copper oxides formed during annealing while the top red layer indicates the adsorption of oxygen from air. The thickness is not to scale. (**d**–**f**) Evolution of the O1s peaks as a function of sputter time. The Cu_2_O peak is at 530.05 eV while the peak of 532.80 eV is attributed to H_2_O^41^,^42^,^43^. (**g**–**i**) Relative atomic contents of O and Cu as a function of sputter time. Using SiO_2_ as a reference, the corresponding thicknesses of oxidation layers for scheme 1–3 are measured to be 60 nm, 3.6 nm and 3 nm, respectively.

To reveal the role of H_2_ in the evolution of single domain Cu(111), the XPS depth-profiling examinations were taken on the Cu samples to measure the thickness of oxide layer along the vertical direction, as illustrated in Fig. 3. Due to the roughness of Cu surface, we use SiO_2_ as a reference instead. The Ar-ion etching rate is about 6 nm min^−1^ based on the calibration of SiO_2_. In scheme 1, the heating and annealing were introduced with Ar flow. Figure 3a shows the schematic structure of a thick oxide layer on Cu(001) substrate. As shown in Fig. 3d, the O1s peaks tend to decrease to zero with increasing the sputter time, indicating that the oxide layer is gradually removed by etching. The thickness of oxide layer on Cu(001) is calculated to be around 60 nm (Fig. 3g). After introducing H_2_ during the heating process in scheme 2, the substrate obtains orientations of Cu(111) and Cu(001) ((Fig. 3b). Two O1s peaks were observed. The peak at B.E. (Binding energy) of 530.05 eV corresponds to Cu_2_O bond^41^, which shrinks quickly by Ar ion etching (Fig. 3e). The smaller peaks at higher B.E. of 532.80 eV correspond to H_2_O bond due to the introduction of H_2_^42^,^43^. According to the disappearance of O1s peak attributed to Cu_2_O bond, the thicknesses of oxide layer in scheme 2 is estimated to be 3.6 nm (Fig. 3h), much thinner than that on the Cu(001) substrate as shown in Fig. 3g. Furthermore, when the heating and annealing were performed under H_2_ flow in scheme 3, the substrate evolves into the single domain Cu(111) orientation (Fig. 3c). Similar with the case in Fig. 3e, the O1s peaks correspond to Cu_2_O bond also decrease sharply by Ar ion etching (Fig. 3f) and the thicknesses of oxide layer on Cu(111) substrate remains nearly 3 nm. In addition, the decreasing oxygen contents from 0.52% (Scheme 1) to 0.44% (Scheme 3) was also observed by the energy dispersive spectroscopy (EDS) (Fig. S9). Combination with the XPS depth-profiling and EDS results, it is believed that the Cu_2_O layer plays a critical role in the orientation transition. With the thickness reduction of Cu_2_O by introducing H_2_, the substrate orientation changes from Cu(001) to Cu(111).

Since the extremely thin Cu_2_O layers were both observed on the mix orientations of Cu(111)/Cu(001) (3.6 nm, scheme 2) and Cu(111) substrate (3 nm, scheme 3), maybe it originates from the air oxidation after the annealed Cu foils were extracted from the CVD chamber. By performing the same XPS depth-profiling examinations, the native oxide layer on the as-received Cu foil is estimated to be 6 nm^44^ (see Fig. S10a,c in Supporting Information), indicating the oxide layers in scheme 2 and scheme 3 derive from the air oxidation^41^. Moreover, in order to prevent the air oxidation, a layer of graphene was deposited on the Cu foil before it was exposed to air in scheme 3, the absence of Cu_2_O peaks in the XPS depth-profiling of as-grown graphene on Cu(111) surface implies that the graphene can play a role in protecting the underlying Cu from oxidation^45^, further demonstrating that the introduction of H_2_ (scheme 3) can completely remove the oxide layer on Cu surface (also see Fig. S10b,d in Supporting Information). It is noted that graphene can protect the surface of Cu substrate from oxidation in a short term due to its impermeability and thermal/chemical stability^45^,^46^. However, the Cu oxidation can still occur^47^,^48^ because the carbon-metal interaction can accelerate electrochemical oxidation of Cu substrate through galvanic corrosion^46^,^49^.

Based on the analysis of XPS depth-profiling, the orientation transition from Cu(001) to Cu(111) in Fig. 1 can be attributed to the thickness reduction of Cu_2_O layer on Cu surface. When the Cu foil is exposed to abundant oxygen, the severe oxidation leads to the smaller grain size of only 5–10 μm. By decreasing the Cu_2_O layer to around 60 nm, the grain size grows up to 50–100 μm. Meanwhile, this kind of Cu_2_O layer tends to stabilize Cu(001) orientation^13^, even annealed at 1040 °C for 180 min (Fig. S3a). Previous work revealed that oxidizing the Cu surface can induce the grain boundary pinning, freezing of the reconstruction of the Cu foil. Subsequent reduction of Cu foil under H_2_, the delayed reconstruction can suddenly be unlocked^23^. As shown in Fig. 1e, once introducing H_2_ to remove the oxide layer in scheme 2, the Cu(111) evolution is driven by the anisotropic force^50^ and there is a grain boundary motion between Cu(111) and Cu(001) planes. After the Cu_2_O layer is completely removed, the Cu substrate changes into the grain boundary-free Cu(111) single domain, as shown in Fig. 1f and Fig. S2. Moreover, once the Cu_2_O layer is removed by H_2_, our results show that the Cu(001) orientation can be transformed into Cu (111) in 1 min, as shown in Fig. S3.

The introduction of H_2_ not only reduces the thickness of Cu_2_O layer, but also reduces the density of nanoparticals generated during the heat treatment and makes Cu surface smooth (Figs S11 and S12), which is consistent with previous reports^15^,^51^. Preheated in Ar only, the Cu surface is rough and is decorated with a high density of nanoparticles. With the introduction of H_2_, the density of nanoparticles is gradually decreasing from scheme 1 to scheme 3, and the roughness of Cu surface is improved obviously. Previous work indicated that the nanoparticles generated during heat treatment can affect the preferential attachment of graphene seeds (Fig. 2a and Fig. S5), where the ordered Cu(111) substrate can control the alignment of graphene islands by strong chemical bonds formed between the graphene edge and atomic step^15^,^52^,^53^,^54^.

The graphene electrical property grown on polycrystalline Cu(001) is obviously inferior to that grown on single domain Cu(111), which can be attributed to three reasons. Firstly, the hexagonal lattice symmetry of Cu(111) surface matches well the honeycomb lattice of graphene (lattice mismatch of ∼4%), enabling epitaxial graphene growth on single domain Cu(111)^10^,^23^,^55^. Secondly, carbon precipitation from the boundaries of Cu(001) grain leads to small elongated islands^56^, as shown in Fig. S7b, degrading the uniformity of graphene. Thirdly, the graphene film on Cu(001) is grown by a lower temperature, leading to the increase of nucleation sites^57^,^58^, as shown in Fig. S7a. The graphene grown on Cu(001) was grown at a relatively low temperature of 900 °C in this work. This is because that the increase of temperature leads to the disappearing of Cu(001) surface as shown in Fig. S3a. At such a low temperature, the graphitization is not as good as at higher temperatures.

**Figure 4:**
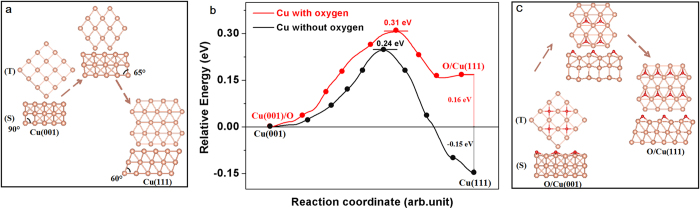
DFT calculations of the effect of oxygen on the orientation transition from Cu(001) to Cu(111). The side (S) and top (T) views of the Cu surface without oxygen (**a**) and the favorable oxygen adsorption on Cu surface (**c**). Note that the oxygen atoms become far away from Cu(111), indicating the weakly bond to the Cu(111) surface. (**b**) The energy barrier between Cu(001) and Cu(111) without and with oxygen are 0.24 eV and 0.31 eV, respectively.

The above analysis clearly shows that the oxygen on Cu surface tends to stabilize the Cu(001) orientation while impedes the evolution of single-domain Cu(111). To further understand the role of O_2_ in crystal orientation control of Cu foils, we conducted the DFT calculations to study the effect of oxygen on the stabilities of Cu(001) and Cu(111), as shown in Fig. 4. Figure 4a illustrates the side and top view of the path for transition from Cu(001) to Cu(111). It can be seen that the marked angles of two structures are changing from 90° to 60°, while a transitional state (the corresponding angle is 65°) is located at the peak of energy barrier. Without considering the oxygen adsorbed on Cu, the surface energy of Cu(111) is 0.15 eV lower than Cu(001), indicating Cu(111) is more stable than Cu(001)^59^. This is the reason why the Cu(111) orientation can be formed once the oxygen is removed by H_2_ flow, as shown in Fig. 1f,i and Fig. S3c–f. Moreover, the energy barrier between Cu(001) and Cu(111) is as high as 0.24 eV, which means a higher annealing temperature is necessary to overcome the barrier^11^,^12^, indicated by Fig. S4a. However, once the attachment of oxygen on Cu surface is considered, the DFT calculation shows that the O/Cu(001) structure becomes 0.16 eV lower than O/Cu(111) structure, indicating O/Cu(001) is more stable than O/Cu(111). Moreover, the energy barrier (0.07 eV) increased by oxygen further impedes the orientation transition from Cu(001) to Cu(111). Our calculation explains the reason why the Cu substrate tends to form Cu(001) orientation once attached to the oxygen atoms, as shown in Fig. 1d,g and Fig. S3a,b.

Understanding the roles of O_2_ and H_2_ in monocrystallization of Cu substrate can be conductive to the achievement of large area Cu(001) or Cu(111) single-crystal, providing an ordered substrate for crystal orientation-controlled synthesis of well-aligned graphene seeds. For example, the introduction of oxygen on Cu surface induced the inch-sized Cu(001) single crystal^13^ while the 12 cm Cu(111) surface was achieved with assistance of hydrogen^11^, which enables the growth of square or hexagonal single-crystal graphene arrays. Considering that the grain boundaries can be suppressed by seamless coalescence of two identical orientations of islands, as demonstrated on the single-crystal Cu(111)^12^, the synthesis of centimeter-size, even meter-size of single-crystalline graphene can be realized.

In summary, we have experimentally and theoretically demonstrated that the oxygen on Cu surface tends to stabilize the Cu(001) orientation while impedes the evolution of Cu(111) single domain. The orientation transition from Cu(001) to Cu(111) is attributed to the thickness reduction of Cu_2_O layer on Cu surface. XPS depth-profiling was performed to measure the thickness of Cu_2_O layer while DFT calculations further reveal that the O/Cu(001) is more stable than O/Cu(111). By removing the Cu_2_O layer on Cu surface, the grain boundary-free Cu(111) single domain can be obtained. The crystal orientation-controlled synthesis of hexagonal graphene arrays has been further realized on the long-range ordered Cu(111). This work provides with a valuable insight into the orientation transition of Cu substrates for growing high-quality graphene films.

## Methods

### Thermal treatments of Cu foils

The as-received commercially available 25 μm thick Cu foils (Kunshan Luzhifa Electron Technology Co., Ltd. China) with a purity of 99.98% were loaded into a home-built CVD system for thermal treatments. Before heating, the purging process was conducted in CVD chamber: Firstly the furnace was evacuated to 0.1 Pa by a vacuum pump, then back filled with high pure Ar until ambient pressure, followed by evacuating to 0.1 Pa again and filling with Ar. This purging process was repeated 3 times to minimize the trapped air in CVD chamber. The detail of 3 schemes: Scheme 1, the sample was heated up to 1040 °C with the flow of 300 sccm of pure Ar then annealed at 1040 °C under the same Ar atmosphere for 30 min; Scheme 2, the sample was heated up to 1040 °C with the flow of 300 sccm of pure H_2_. After reaching 1040 °C, the H_2_ was turned off and the Cu foil was annealed under 300 sccm of pure Ar for 30 min; Scheme 3, the sample was heated up to 1040 °C with 300 sccm of pure H_2_ then annealed at 1040 °C under the same H_2_ atmosphere for 30 min. The gas atmosphere, temperature, pressure, and cooling rate were carefully controlled by a CVD system.

### Graphene growth and transfer

After thermal treatments of Cu foils, the furnace was maintained at the same temperature (900 °C for graphene growth on Cu(001) and 1040 °C for graphene on Cu(111)). A small amount of CH_4_ (the concentration was diluted to 0.03–0.05% by H_2_-Ar mixture gas) was introduced into the CVD reactor at ambient pressure for 0.5–5 min. Finally the CVD furnace was cooled down to room temperature at a rate of 0.3–0.9 °C/s after the heater power was shut down. After growth, graphene films grown on Cu(111) and Cu(001) substrates were transferred to target substrates using the typical PMMA-mediated transfer-printing technique. All the data was collected for samples with two desired growth temperatures 900 °C and 1040 °C. Figure 2 data has been taken on graphene grown on Cu(111) at 1040 °C; Fig. S4 data has been taken on graphene grown on Cu(001) at 900 °C.

### Characterizations

X-ray diffractometer (German, D8 Advance) and SEM (CamScan, Apollo 300) equipped with EBSD (HKLNordlysNano, Oxford) were used to analyze the macrotexture of Cu substrate. X-ray photoelectron spectroscopy (XPS,ULVAC-PHI 5000 Versaprobe II) and EDS mapping (Quanta 250 FEG, USA) were used to identify the surface elements of Cu substrates. The morphology of Cu surface was measured by Atomic Force Microscope (Dimension 3100) at a scan rate of 1.2 Hz and 512 × 512 resolutions. The surface morphology of the transferred graphene film was investigated through Scanning Electron Microscope (Quanta 400 FEG, USA) and Optical Microscope (Olympus BX51, Japan). The optical transmittance of graphene/Cu(001) and graphene/Cu(111) films with an area of 10 mm × 10 mm on glass was determined through UV-vis Spectroscopy (Perkin Elmer Lambda 25, USA). Raman Spectrometer (LabRam HR800-UV-NIR, λ = 532.15 nm, France) was used to identify the layer number and defect density of graphene/Cu(001) and graphene/Cu(111) films. The sheet resistance maps (50 points in an area of 15 mm × 15 mm) of graphene films were obtained via Semi-automatic 4 probe measurement system (CRESBOX, NAPSON) based on the van der Pauw method.

### Device fabrication and transport measurements

The carrier mobilities of graphene grown on Cu(001) and Cu(111) substrates were extracted from the back-gated field-effect-transistors (FETs) devices, with channel length (*L*_*C*_) 50 μm and channel width (*L*_*W*_) 50 μm fabricated by standard photolithography technique. The electrical measurements of the devices were carried out at room temperature with the aid of Agilent B1500A semiconductor parameter analyzer. The gate voltage was applied through the doped Si substrate and the source-drain bias (V_ds_) was constant at 0.01 V. The mobility was estimated by using the formula:


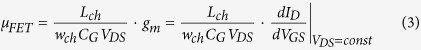


where *L*_*ch*_ and *W*_*ch*_ are the channel length and channel width, respectively. *C*_*G*_ is the gate oxide capacitance per unit area, *I*_*D*_ is the source–drain current, *V*_*GS*_ is the gate voltage and *V*_*DS*_ is the source–drain voltage.

### DFT calculations

To understand the transition mechanism, we search for the minimum energy pathway (MEP) between phases utilizing transition state theory as formulated in the generalized solid-state nudged elastic band (G-SSNEB) method implemented in the VTST extension of the Vienna ab initio simulation package (VASP). The calculations employed the projector augmented wave (PAW) method and the generalized gradient approximation (PBE-GGA) for electron exchange-correlation interaction. The energy cutoff for the plane wave functions is 460 eV with 7 × 7 × 1 Γ -centered Monkhorst-Pack grids, and a force acting on each atom of <0.001 eV/Å was used as the criterion of convergence in geometrical optimization. The Cu surface is modeled using a supercell approach, where we use a six-layered Cu slab with a vacuum region of 25 Å. Oxygen atoms are adsorbed on both sides of the slab, preserving inversion symmetry.

## Additional Information

**How to cite this article:** Hu, J. *et al*. Roles of oxygen and hydrogen in crystal orientation transition of copper foils for high-quality graphene growth. *Sci. Rep.* **7**, 45358; doi: 10.1038/srep45358 (2017).

**Publisher's note:** Springer Nature remains neutral with regard to jurisdictional claims in published maps and institutional affiliations.

## Supplementary Material

Supplementary Information
