# Supplementary material for: Roles of Oxygen and Hydrogen in Crystal Orientation Transition of Copper Foils for High-Quality Graphene Growth
Source: Sci Rep. 2017 Apr 3;7:45358. doi: 10.1038/srep45358 (PMC5377254; doi:10.1038/srep45358)
Supplement: Supplementary Information [file srep45358-s1.doc]

**Supporting information**

Roles of Oxygen and Hydrogen in Crystal Orientation Transition of Copper Foils for High-Quality Graphene Growth

Junxiong Hu^1,2,*^, Jianbao Xu^1,3,*^, Yanfei Zhao^4^, Lin Shi^5^, Qi Li^1^, Fengkui Liu^1^, Zaka Ullah^1^, Weiwei Li^1,6^,, Yufen Guo^1,6^, & Liwei Liu^1^

^1^Key Lab of Nanodevices and Applications, Suzhou Institute of Nano-Tech and Nano-Bionics, Chinese Academy of Sciences (CAS), Suzhou 215123, P. R. China.

^2^Department of Physics, Institute of Low-dimensional Carbons and Device Physics, Shanghai University, Shanghai 200444, P. R. China.

^3^College of Materials Sciences and Opto-Electronic Technology, University of Chinese Academy of Sciences, Beijing 100049 , P. R. China.

^4^Nano-X, Suzhou Institute of Nano-Tech and Nano-Bionics, Chinese Academy of Sciences (CAS), Suzhou 215123, P. R. China.

^5^Platform for Characterization &Test, Suzhou Institute of Nano-Tech and Nano-Bionics, Chinese Academy of Sciences (CAS), Suzhou 215123, P. R. China.

^6^Suzhou Graphene Nanotechnology Co.,Ltd. Suzhou 215123, P. R. China.

^＊^These authors contributed equally to this work. Correspondence and requests for materials should be addressed to L.L. (email: **lwliu2007@sinano.ac.cn).**


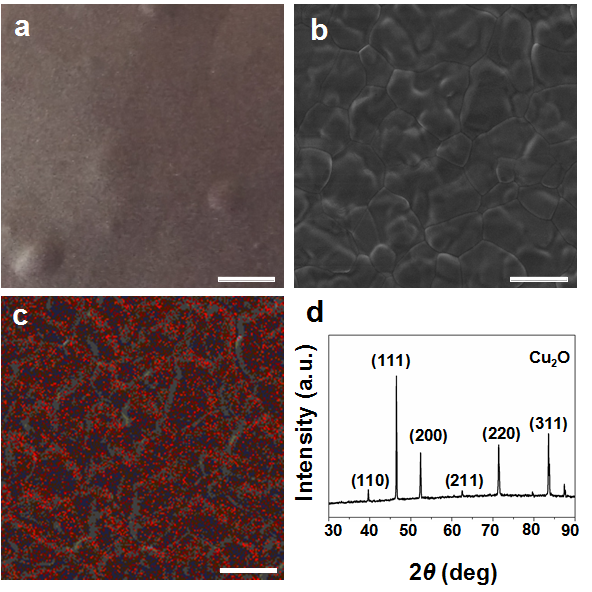


**Supplementary Figure S1. Surface morphology and structure characterizations of Cu foils annealed without conducting purging process. (a)** Photograph of Cu foil after annealing. The Cu foil becomes completely black due to the severe oxidation. scale bar, 1 cm. **(b)** SEM image of Cu foil showing small grain size. scale bar, 5 µm. **(c)** EDS mapping of O element for the sample from **(b)**, the O content is high as 7.2% confirmed from EDS spectrum. Scale bar, 5 µm. **(d)** XRD profile of Cu foil. The peaks are analyzed for Cu_2_O. The condition: without conducting purging process, the Cu foil was directly heated to 1040 ℃ under Ar flow within 40 min, then annealed at 1040 ℃ under the same Ar atmosphere for 30 min.

**
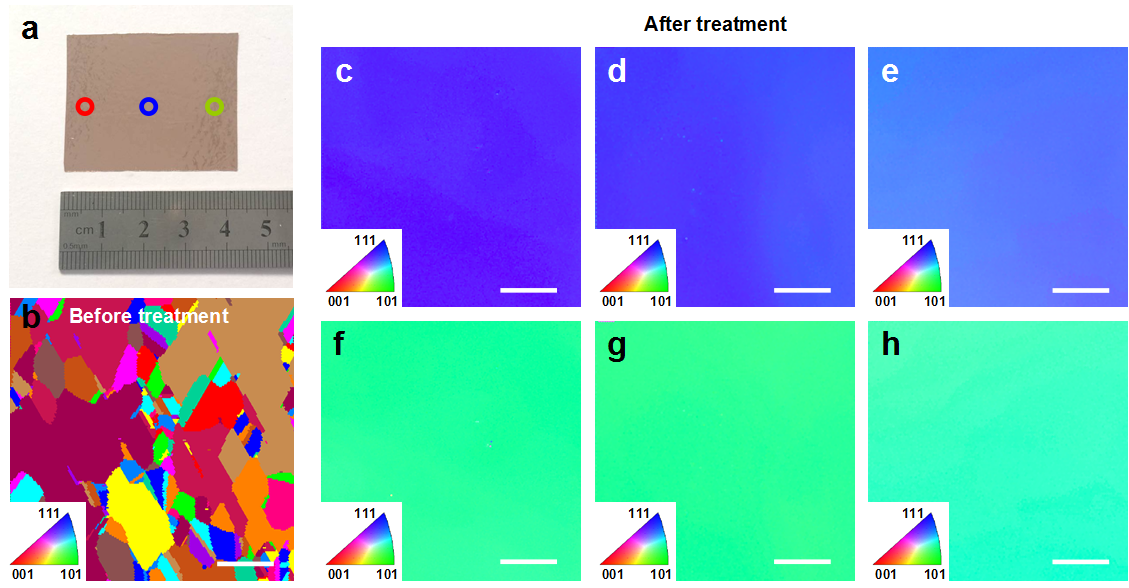
**

**Supplementary Figure S2. EBSD images of Cu foil annealed under the scheme 3. (a)** Photograph of large-area Cu foil (4 cm × 3 cm) after heat treatment, with three colored circles locating where the maps are recorded. **(b)** EBSD map of Cu foil before heat treatment. EBSD out-of-plane **(c-e)** and in-plane **(f-h)** inverse pole figure maps at the three locations, with a frame colored according to the circles in **(a).** Scale bars, 200 μm **(b-h)**. The corresponding inverse pole figure is in inset to each inverse pole figure map. The uniform color in each out-of-plane map confirms the large-scale Cu(111) reconstruction of the Cu substrate while the uniform color in each in-plane map shows there is no rotational misfit between the three spots, so that the marked three areas belong to the same Cu crystal.

**
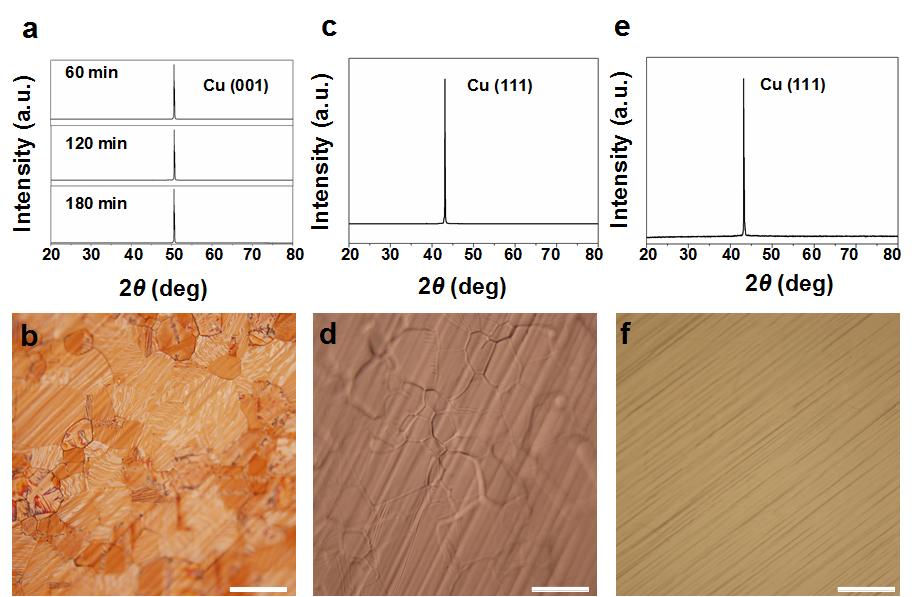
**

**Supplementary Figure S3. Structure characterization of the Cu foils annealed without and with hydrogen. (a)** XRD profiles of Cu foils annealed under Ar atmosphere. The condition: after conducting the cleaning process, the Cu foils were heated and annealed at 1040 ℃ under ambient pressure with 300 sccm of pure Ar for 60,120, and 180 min. **(b)** The optical image of corresponding Cu foil annealed under Ar atmosphere for 180 min. Scale bar, 50 µm. **(c)** XRD profile of Cu foil annealed under Ar and H_2_ atmosphere. The condition: the Cu foil firstly annealed at 1040℃ under ambient pressure with 300 sccm of Ar for 180 min, followed by annealing at 300 sccm of H_2_ for 1 min. Scale bar, 50 µm. **(d)** The optical image of Cu foil from **(c). (e)** XRD profile of Cu foil annealed under H_2_ atmosphere. The condition: the Cu foil annealed at 1040℃ under ambient pressure with 300 sccm of pure H_2_ for 1min. **(f)** The optical image of Cu foil from **(e).** Scale bar, 50 µm.


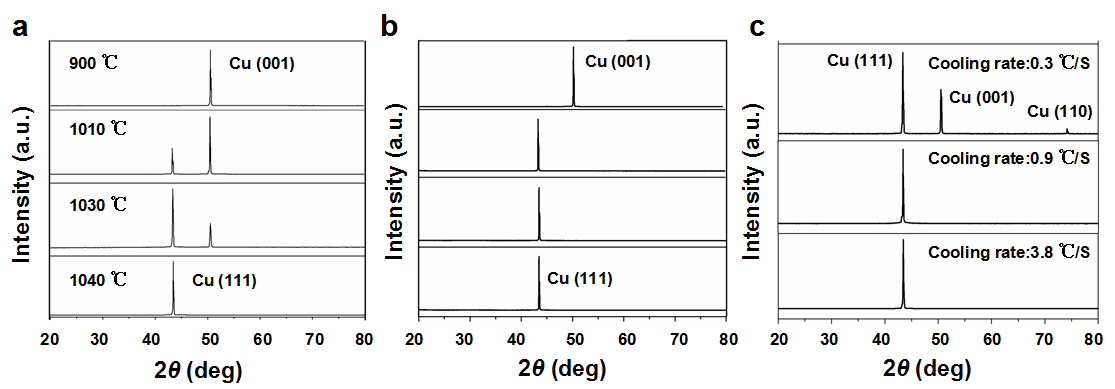


**Supplementary Figure S4. Effects of temperature, pressure and cooling rate on orientation transition from Cu(001) to Cu(111).** XRD profiles of Cu foils annealed at elevating temperatures 900 ℃, 1010 ℃,1030 ℃, 1040 ℃ **(a)** under ambient-pressure with 300 sccm of H_2_ for 30 min and **(b)** under low-pressure at 150 Pa with 300 sccm of H_2_ for 30 min. **(c)** XRD profiles of Cu foils under different cooling rates after annealed at 1040 ℃ with 300 sccm of H_2_ for 30 min.


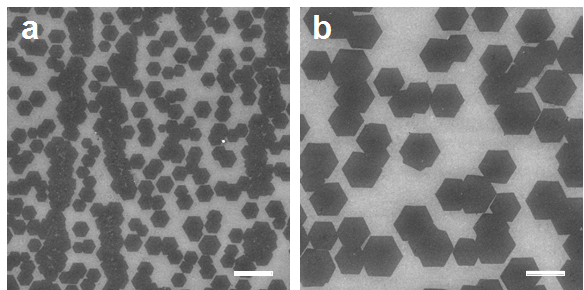


**Supplementary Figure S5.** **Characterization of graphene grown on single domain Cu(111) at the early-stage growth.** SEM images of early-stage graphene growth (with increasing magnification from **(a)** to **(b)**; the scale bars correspond to 40 µm, and 20 µm respectively), showing extended linear arrays of well-aligned hexagonal domains.


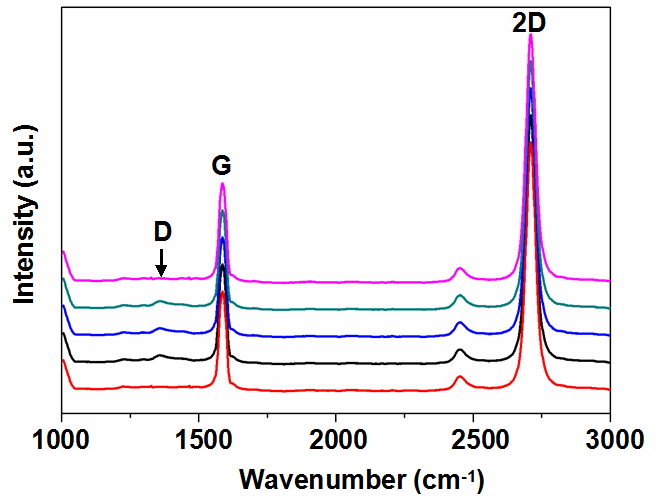


**Supplementary Figure S6.** Five Raman spectra recorded at random positions on the sample. Note that the spectra are shifted vertically relatively to each other for clarity.

**
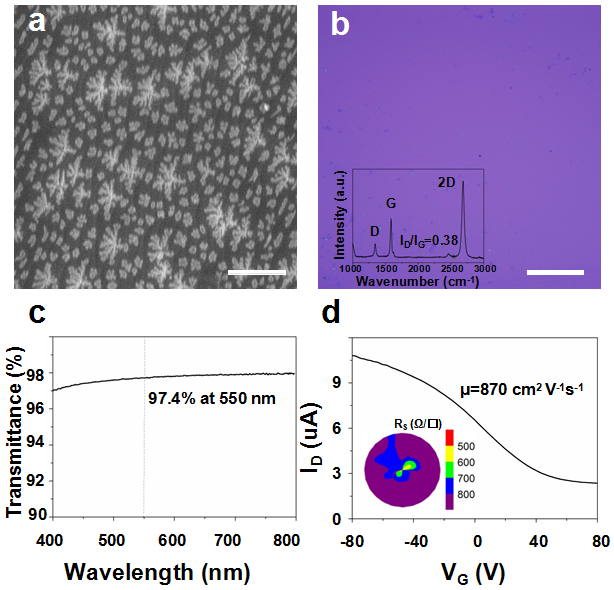
**

**Supplementary Figure S7. Optical and electrical characterizations of graphene grown on polycrystalline Cu(001) substrate. (a)** SEM image of a partial-coverage graphene seeds on SiO_2_/Si. Scale bar, 2 µm. **(b)** Optical image of continuous graphene film, the inset shows a typical Raman spectrum of graphene films transferred onto SiO_2_/Si. Scale bar, 20 µm. **(c)** UV-vis transmittance spectrum of graphene film transferred to the glass, showing a single layer graphene. **(d)** Current−voltage (I_D_−V_G_) curve for the continuous graphene grown on Cu(001)-FET measured at room temperature, the left inset shows the sheet resistance distribution of graphene film transferred onto SiO_2_/Si(1.5 mm in diameter).


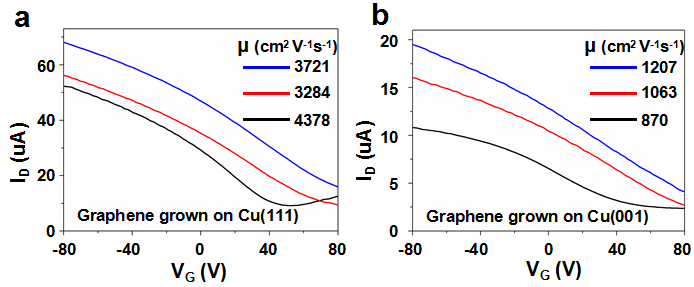


**Supplementary Figure S8.** Current−voltage (*I_D_−V_G_*) curves of the three different transistors for the continuous graphene grown on **(a)** Cu(111) and **(b)** Cu(001) measured at room temperature. The carrier mobility of graphene grown on Cu(111) is obviously higher than that of graphene grown on Cu(001).

**
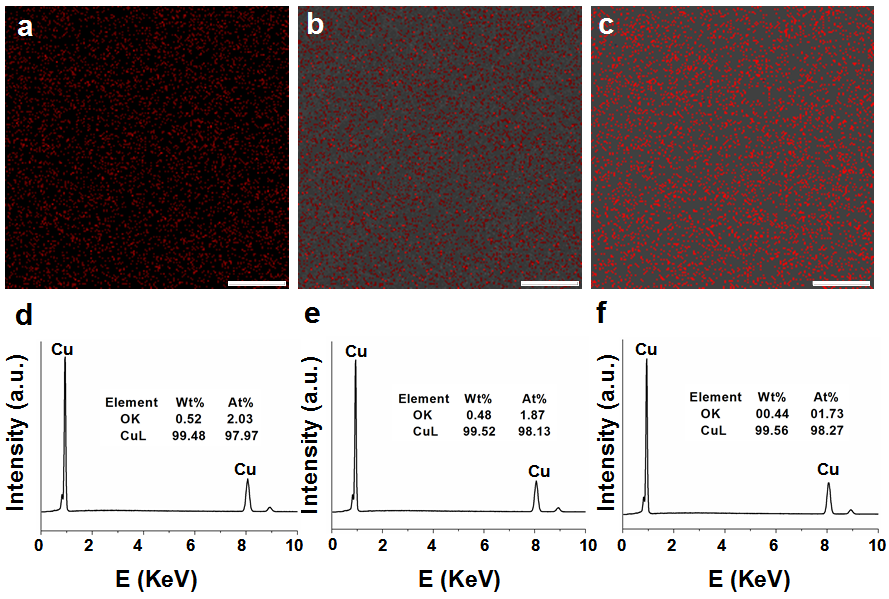
**

**Supplementary Figure S9. EDS analysis of the Cu foils annealed under 3 schemes. (a-c)** The EDS mappings of O element for Cu foils. Scale bars, 50 µm. **(d-f)** EDS spectra are recorded on the area (200 µm × 200 µm) shown in **(a-c)**. Note that only Cu and O elements are detected and the oxygen contents tend to decrease from 0.52% ( scheme 1) to 0.44% ( scheme 3).

**
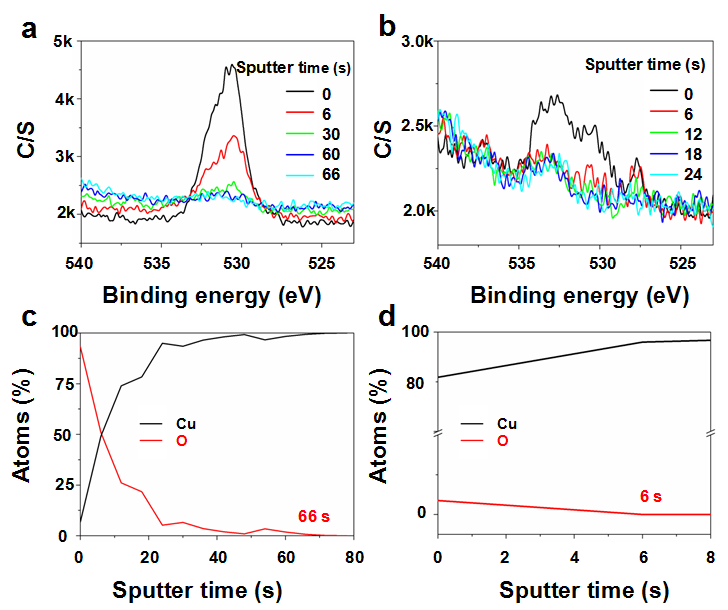
**

**Supplementary Figure S10. XPS analysis of as-received Cu foil (a,c) and graphene-Cu foil (b,d). (a,b)** Evolution of the O peaks as a function of the sputter time. Note that the peaks of 530.05 eV in **(a)** are assigned to Cu_2_O while the peaks of 532.8 eV in **(b)** arose from H_2_O. **(c,d)** Relative atomic contents of O and Cu as a function of sputter time. Using SiO_2_ as a reference, the corresponding thicknesses of oxide layers for as-received Cu foil and graphene-Cu foil were measured to be 6.6 nm and 0.6 nm, respectively.


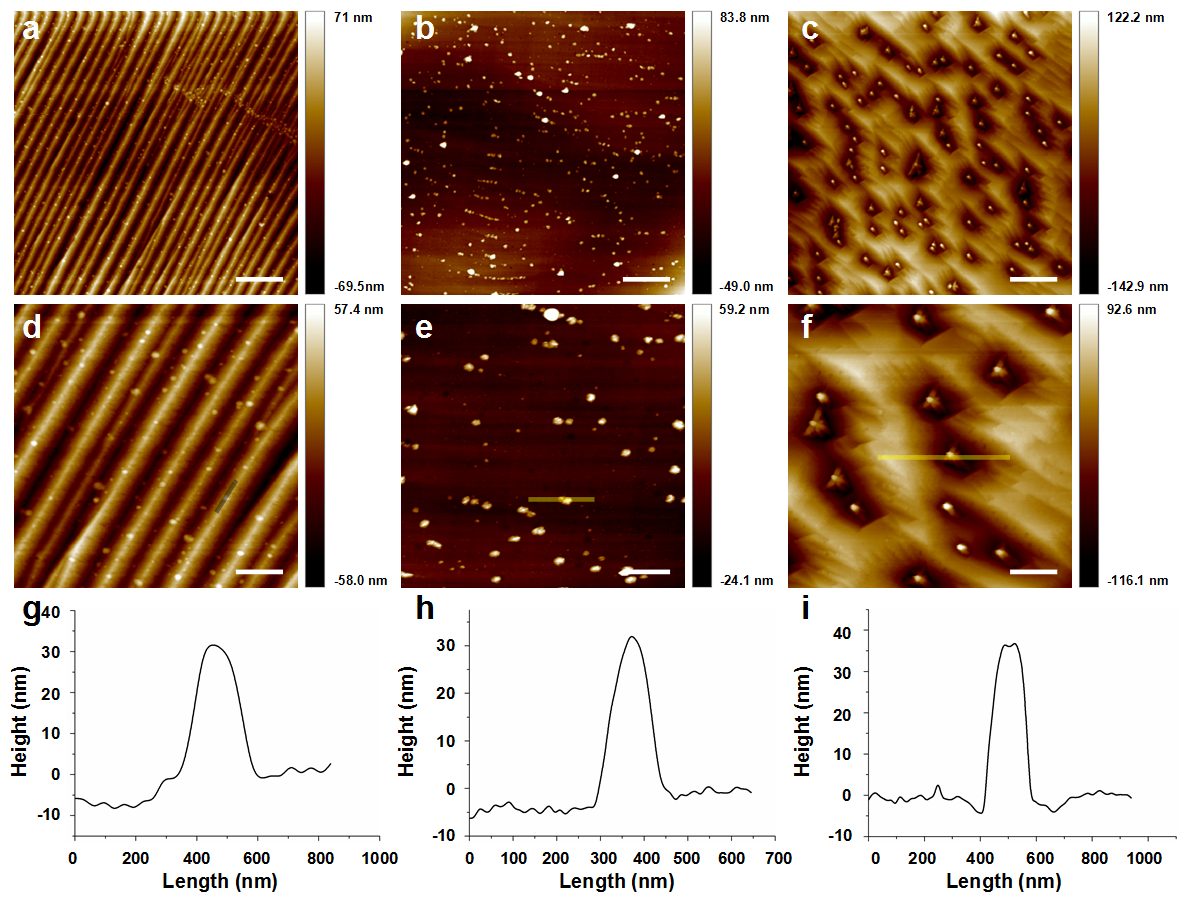


**Supplementary Figure S11. AFM images of Cu foils annealed under 3 schemes.** **(a,d)** heating and annealing under Ar flow. **(b,e)** heating and annealing under H_2_ and Ar flows, respectively. **(c,f)** heating and annealing under H_2_ flow. Heating and annealing in Ar resulted in a rough surface decorated with a large number of nanoparticles, while heating and annealing in H_2_ results in a rather low roughness surface and low density of nanoparticles. Scale bars, 3 μm **(a-c)**; 1 μm **(d-f)**.

**
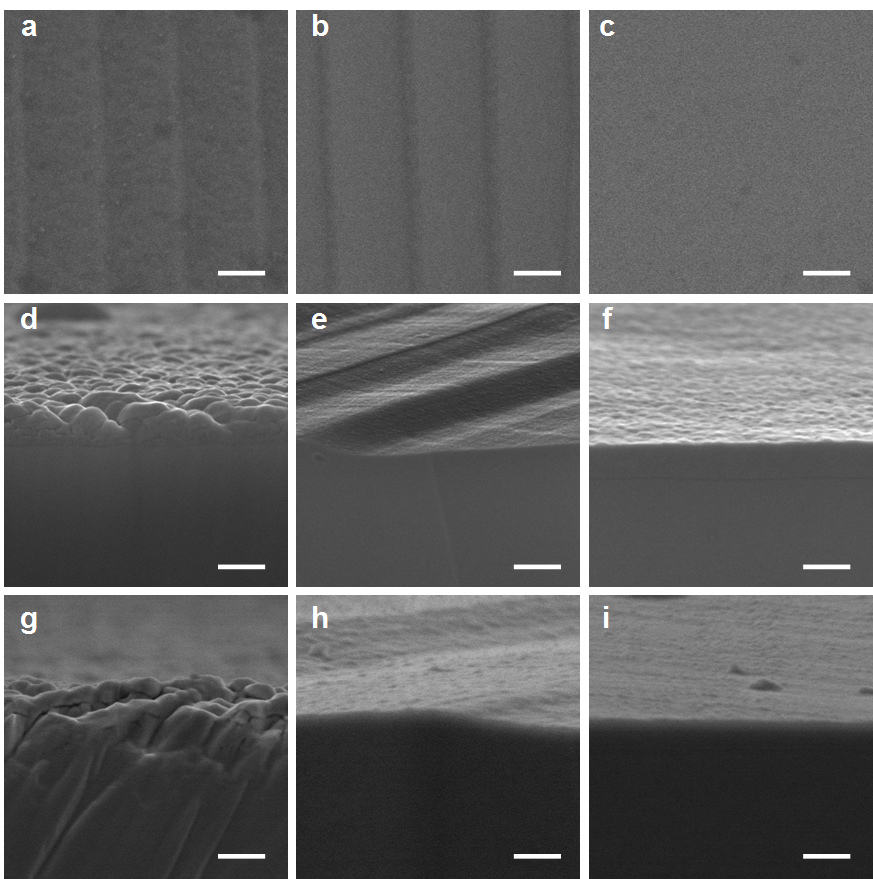
**

**Supplementary Figure S12. SEM images of top and side views of Cu foils annealed under 3 schemes.** (a,d,g) Heating and annealing under Ar flow. (b,e,h) Heating and annealing under H_2_ and Ar flows, respectively. (c,f,i) Heating and annealing under H_2_ flow. Scale bars: 200 nm. The images of top (a-c) and side views (d-f) of Cu foils taken by the thermal field SEM. The images of side views (g-i) of Cu foils taken by the cold field SEM. With the introduction of H_2_ from scheme 1 to scheme 3, the surface of Cu foil shows a rather low roughness.
